# Effects of geohelminth infection and age on the associations between allergen-specific IgE, skin test reactivity and wheeze: a case-control study

**DOI:** 10.1111/cea.12040

**Published:** 2012-12-24

**Authors:** A-L Moncayo, M Vaca, G Oviedo, L J Workman, M E Chico, T A E Platts-Mills, L C Rodrigues, M L Barreto, P J Cooper

**Affiliations:** 1 Instituto de Saude Coletiva, Universidade Federal da BahiaSalvador, Bahia, Brazil; 2 Colegio de Ciencias de la Salud, Universidad San Francisco de QuitoQuito, Ecuador; 3 Asthma and Allergic Diseases Center, University of VirginiaCharlottesville, VA, USA; 4 Department of Epidemiology, London School of Hygiene and Tropical MedicineLondon, UK; 5 Molecular and Biochemical Parasitology, Liverpool School of Tropical MedicineLiverpool, UK

**Keywords:** allergen skin test reactivity, allergen-specific IgE, atopy, geohelminths, wheeze

## Abstract

**Background:**

Most childhood asthma in poor populations in Latin America is not associated with aeroallergen sensitization, an observation that could be explained by the attenuation of atopy by chronic helminth infections or effects of age.

**Objective:**

To explore the effects of geohelminth infections and age on atopy, wheeze, and the association between atopy and wheeze.

**Methods:**

A case-control study was done in 376 subjects (149 cases and 227 controls) aged 7–19 years living in rural communities in Ecuador. Wheeze cases, identified from a large cross-sectional survey, had recent wheeze and controls were a random sample of those without wheeze. Atopy was measured by the presence of allergen-specific IgE (asIgE) and skin prick test (SPT) responses to house dust mite and cockroach. Geohelminth infections were measured in stools and anti-*Ascaris* IgE in plasma.

**Results:**

The fraction of recent wheeze attributable to anti-*Ascaris* IgE was 45.9%, while those for SPT and asIgE were 10.0% and 10.5% respectively. The association between atopy and wheeze was greater in adolescents than children. Although Anti-*Ascaris* IgE was strongly associated with wheeze (adj. OR 2.24 (95% CI 1.33–3.78, *P* = 0.003) and with asIgE (adj. OR 5.34, 95% CI 2.49–11.45, *P* < 0.001), the association with wheeze was independent of asIgE. There was some evidence that the association between atopy and wheeze was greater in uninfected subjects compared with those with active geohelminth infections.

**Conclusions and clinical relevance:**

Atopy to house dust mite and cockroach explained few wheeze cases in our study population, while the presence of anti-*Ascaris* IgE was an important risk factor. Our data provided only limited evidence that active geohelminth infections attenuated the association between atopy and wheeze in endemic areas or that age modified this association. The role of allergic sensitization to *Ascaris* in the development of wheeze, independent of atopy, requires further investigation.

## Introduction

The International Study of Asthma and Allergies in Childhood (ISAAC) studies have shown that childhood asthma defined by the presence of recent wheeze is extremely common in many Latin America countries, where the prevalence is as high as in industrialized countries such as UK and US 1. In addition, asthma prevalence is consistently higher in poor populations in urban areas in Latin America 2, 3 compared with rural areas 4, 5. Possible explanations for these findings include differences in environmental exposures associated with urbanization and the acquisition of a ‘modern’ lifestyle 1.

Asthma and atopic diseases show large differences by age in incidence, prevalence and severity 6. Asthma starts during the first years of life 7, while the incidence of hay fever generally peaks around school age and adolescence in industrialized countries 8. Sensitization to aeroallergens increases during childhood and adolescence, reaching a peak in the third decade of life 9, 10. A more persistent phenotype of asthma has been associated with atopy in late childhood and early adolescence that may continue into adulthood, while early onset asthma in the absence of atopy may be more likely to resolve 6, 7. Environmental triggers of atopic diseases may mediate their effects during discrete time windows such as the first 2 years of life and exposure to these triggers may vary by age resulting in time and age-dependent changes in disease incidence 11, 12. Thus, the relationship between asthma and atopy may differ between childhood and adolescence being associated with age-specific differences in environmental exposures and the hormonal changes of puberty 11.

Atopy can be measured either by the presence of skin prick test (SPT) reactivity or allergen-specific IgE (asIgE) in serum to common aeroallergens 13. SPT is often preferred in large epidemiological studies because it is easily performed, provides an immediate result, and is commonly used to validate *in vitro* tests for specific IgE 13. However, the results of SPT and asIgE are strongly dissociated in some populations particularly those living in developing countries, where a significant proportion of those with asIgE do not have SPT to the same aeroallergens 14–17. This observation has been attributed to a down-regulation of allergic effector responses associated with environmental exposures such as infections with helminth parasites 1.

The link between asthma and atopic sensitization increases with economic development 14. In the ISAAC phase II study, the population fraction of asthma attributable (PAF) to atopy measured by SPT was 41% in ‘affluent’ countries but only 20% in ‘non-affluent’ countries in children aged 8–12 years 14. Similar results were obtained when the presence of specific IgE to common aeroallergens (asIgE) was used to define atopy (45.6% vs. 18.3% respectively) 14 Moreover, in the two Latin American study centres included in the ISAAC Phase II study (Pichincha Province, Ecuador and Uruguaiana, Brazil), only 11% of asthma was attributable to SPT 14, while a study of children of the same age living in rural Esmeraldas Province in Ecuador showed that only 2.4% of asthma was attributable to SPT 18. The weak associations between asthma and atopy in such populations could be explained by the attenuation of atopy or Th2-mediated allergic responses by environmental factors including chronic helminth infections 19, 20.

To investigate the effects of geohelminth infections and age on atopy, wheeze, and the association between atopy and wheeze, we conducted a case-control study of recent wheeze in school-age children and adolescents living in a rural area of tropical Ecuador.

## Methods

### Study area and population

The study was conducted among school children and adolescents in rural Afro-Ecuadorian communities in the districts of Eloy Alfaro and San Lorenzo, in Esmeraldas province, Northeastern Ecuador. The characteristics of the study area and population have been described in detail elsewhere 21.

### Study design

Between March 2005 and May 2007, a cross-sectional survey was conducted among 3960 school children between 6 and 16 years of age 18. Within this study population, a nested case-control study was performed between November 2007 and March 2009, with wheeze cases selected from the population based on the presence of recent wheeze and controls as a random sample of those without symptoms. Individuals considered eligible to be cases were those with a positive (parental) response to the question, ‘Has your child had wheeze in the chest in the last 12 months?’ (‘El niño(a) ha tenido silbido al pecho en los ultimos 12 meses?’), in the cross-sectional survey and eligible controls were those individuals with a negative response to the question, ‘Has your child ever had wheeze in the chest? (Alguna vez en la vida, el niño(a) tuvo silbido al pecho (en cualquier epoca del pasado?)’. At the time of selection of cases and controls, a brief screening questionnaire was administered again to parents. Cases were those children for whom a second positive parental response was obtained to the first question. Four controls for each case were selected from random lists generated using Stata 9 software (Statacorp, College Station, TX, USA) and were eligible if a second negative response was obtained to the second question. Individuals with inconsistent responses for the two questions in the two questionnaires were excluded. The time between the cross-sectional and screening questionnaires was approximately 2 years.

### Sample size and study power

A pilot study estimated the prevalence of wheeze within the previous 12 months and SPT in rural area as being 7.3% and 13.5% respectively. We estimated that a sample of 4000 children in the rural area would be needed to obtain 292 wheeze cases or 200 cases with losses of 30% and 800 controls (case-control ratio 1 : 4) to detect an effect on wheeze of OR < 0.6 for common exposures (40–60% prevalence – e.g. geohelminth infections and household pets), and OR < 0.4 for rare exposures (10% – e.g. family history of allergic disease) with a study power of 80% and *P* < 0.05 21.

### Questionnaire

A questionnaire was administered to the child's parent or guardian in the presence of the child that included the core allergy questions of the ISAAC phase II study 22 and supplementary questions, including a detailed history of asthma, rhinitis and eczema symptoms, the management of asthma and potential risk factors 21, 22.

### IgE measurements

Blood samples (7 mL) were collected by venipuncture from participants, centrifuged and plasma was stored at – 20°C until testing. Total IgE and IgE antibodies specific for *Dermatophagoides pteronyssinus, Periplaneta americana* (American cockroach) and *Ascaris lumbricoides* were measured using the Pharmacia CAP system (Phadia AB, Uppsala, Sweden) according to the manufacturer's instructions. Measurements of asIgE were restricted to *D. pteronyssinus* and *P. americana*. We analysed asIgE in three ways: (1) asIgE level: the highest concentration of asIgE observed for either mite or cockroach; (2) asIgE category: the highest concentration of asIgE observed for either mite or cockroach within the following ranges, < 0.70, ≥ 0.70–3.49, and ≥ 3.5 kU/L; (3) asIgE positivity: having any asIgE ≥ 0.70 kU/L. Levels of total IgE were log_e_-transformed and expressed as geometric means.

### Allergen skin prick testing

Allergic sensitization was measured by skin prick testing with seven allergen extracts (Greer laboratories, Lenoir, NC, USA): *D. pteronyssinus/farinae* mix, American cockroach (*P. americana*), *Alternaria tenuis*, cat dog, ‘9 southern grass mix’ and ‘New stock fungi mix’, positive histamine and negative saline controls. A positive reaction was defined as a mean weal diameter at least 3 mm greater than the saline control 15 min after pricking the allergen onto the volar side of the forearm using ALK lancets (ALK, Hungerford, UK). For the purposes of analysis, only those children with positive SPTs to cockroach and mite were considered to be SPT positive.

### Stool examinations

Single stool samples were collected and analysed for geohelminth eggs and larvae using the modified Kato Katz (quantification of *A. lumbricoides* and *Trichuris trichiura*) and formol-ether concentration (detection of all geohelminths including hookworm and *Strongyloides stercoralis*) methods 23. As stool eggs counts were over-dispersed, they were log_e_-transformed and expressed as geometric means.

### Exercise-induced bronchospasm

Each child underwent a vigorous six-minute free running exercise with spirometry performed before and five minutes after exercise as described previously 21. The highest forced expiratory volume in 1 s (FEV_1_) from five efforts before and after the exercise were used to measure exercise-induced bronchospasm (EIB) as follows: [(Pre-exercise FEV_1_ − Post-exercise FEV_1_)/(Pre-exercise FEV_1_)] × 100. For the purposes of this study, EIB was defined as a fall of 10% in FEV_1_ after exercise. The heart rate before and after exercise was recorded as a measure of the intensity of the exercise.

### Statistical analysis

Differences in the prevalence of asIgE (≥ 0.70 kU/L), SPT and geohelminth infection markers between cases and controls were evaluated using *χ*^2^ or Fisher's exact test where appropriate. Population attributable fractions (PAFs) were calculated using the formula P × (OR − 1)/OR where P is the prevalence of SPT or asIgE among children with recent wheeze. We evaluated the effects of age [childhood (7–11 years) vs. adolescence (12–19 years)] with the cut-off defined by the median age of 12 years on atopy and wheeze as follows: (1) A Venn diagram was used to display the distribution of asIgE and SPT responses in asthmatic cases and non-asthmatic controls by age group; and (2) the associations of asIgE, SPT and anti-*Ascaris* IgE with wheeze were estimated for each of the two age groups using random effects logistic regression allowing adjustment for clustering. ORs were adjusted for sex, maternal allergic diseases and maternal smoking.

Geohelminth infections or exposures may act as effect modifiers of the associations between markers of atopy and wheeze. The potential effects of markers of geohelminth infection (positive stool samples for *A. lumbricoides* and/or *T. trichiura* and presence of anti-*Ascaris* IgE [≥ 0.7 kU/L) on the following associations were assessed using random effects logistic regression or logistic regression models with the ‘survey’ estimation (to take in account the effect of case-control design and two-level data structure) where appropriate: (1) SPT and recent wheeze; (2) asIgE and recent wheeze; and (3) asIgE and SPT. Models were built separately for presence or absence of geohelminth infections in stools and for presence or absence of anti-*Ascaris* IgE. We used hierarchical analyses to explore whether associations between anti-*Ascaris* IgE and wheeze might be explained by asIgE (i.e. by treating asIgE as an intermediate variable in the association between anti-*Ascaris* IgE and wheeze). For this analysis, the ORs for the models that exclude and include asIgE are compared – if inclusion reduces the OR towards 1 then this suggest that part of the association may be explained by asIgE. In addition, we evaluated the effects of the two geohelminth infection markers on wheeze, asIgE, and SPT and their joint effects through stratification into four groups: (1) no active infection or IgE sensitization to *Ascaris* (geohelminth ova negative and absence of anti-*Ascaris* IgE), (2) active infection without IgE sensitization to *Ascaris* (ova positive and absence of anti-*Ascaris* IgE), (3) no active infection but evidence of IgE sensitization to *Ascaris* (ova negative and presence of anti-*Ascaris* IgE), (4) active infection in the presence of IgE sensitization to *Ascaris* (ova positive and presence of anti-*Ascaris* IgE). Atopic wheeze was defined by wheeze within the previous 12 months in the presence of asIgE (≥ 0.70 kU/L) to either house dust mite and or cockroach. Multivariate multinomial logistic regression was used to estimate the association between the markers of geohelminth infection and wheezing among non-atopic children (by comparing non-atopic wheezers with non-atopic non-wheezers) and for wheezing among atopic children (by comparing atopic wheezers with atopic non-wheezers). All models were adjusted for *a priori* confounders of age, sex, maternal allergic diseases and maternal smoking (for wheeze outcomes only). Analyses stratified by exposure levels for which separate ORs were estimated were adjusted for *a priori* confounders only because of issues of convergence. Overall analyses for study outcome-exposure associations were adjusted for other confounders using a backward stepwise procedure in which individual covariates were kept in the models if they were significant predictors of the outcome (at *P* < 0.05) or if they altered OR for the outcome by > 10%. The potential confounders considered were body mass index, day care attendance, household pet exposures, breastfeeding, sedentarism (exercise levels and time spent watching TV), maternal educational level, source of drinking water, excreta disposal and crowding. All analyses were done using STATA, version 10 (Statacorp).

### Ethics

The study was approved by the ethics committee of the Hospital Pedro Vicente Maldonado, Ecuador (9-12-04), and the study was conducted in accordance with the principles expressed in the Declaration of Helsinki. Written informed consent was obtained from the parent of each child and signed minor assent from the child. The parent or guardian of each child was provided with a copy of all laboratory results, and all children with geohelminth infections were offered appropriate treatment.

## Results

### Characteristics of study population

A total of 839 children (154 cases and 685 controls) were recruited in the case-control study. The data included in the present analysis were from a sample of 379 of these children for whom measurement were done of asIgE, total IgE and anti-*Ascaris* IgE in plasma. This sample included 150 (97.4%) of the 154 asthma cases and a random sample of controls [229/685 (33.4%)]. The number of children with complete data for recent wheeze and markers of atopy was 376 (149 cases and 227 controls) and with data for geohelminth infections also was 363 (142 cases and 221 controls). The characteristics of cases and controls are shown in Table 1. Among infected children, the geometric mean infection intensity for *A. lumbricoides* was 2587 eggs per gram (epg) of feces (range, 70–169, 400) and 785 epg (range, 70–83, 650) for *T. trichiura,* and did not vary between cases and controls. Geometric mean levels of total IgE (668 vs. 427 IU/mL, *P* = 0.002) and specific IgE for *Ascaris* (2.96 vs. 1.45 kU/L, *P* < 0.0001) and *D. pteronyssinus* (0.72 vs. 0.50 kU/L, *P* = 0.003) were significantly greater in cases than controls, but there were no differences in levels of specific IgE for *P. americana* (0.50 vs. 0.47 kU/L, *P* = 0.41). The PAFs for recent wheeze associated with detectable levels of asIgE and SPT were 10.5% and 10.0%, respectively, while that for anti-*Ascaris* IgE was 45.9%.

**Table 1:** Characteristics of cases and controls

	Cases	Controls	*P*-value*
Variables	*n* = 149	*n* = 227	
Age (years)			
7–10	59 (39.6%)	56 (24.7%)	
11–14	70 (47.0%)	110 (48.5%)	
15–19	20 (13.4%)	61 (26.9%)	0.001
Sex			
Male	83 (55.7%)	129 (56.8%)	
Female	66 (44.3%)	98 (43.2%)	0.830
Maternal education level [5]			
Complete secondary or higher	14 (9.4%)	16 (7.2%)	
Complete primary or incomplete secondary	55 (36.9%)	94 (42.3%)	
Illiterate or incomplete primary	80 (53.7%)	112 (50.5%)	0.506
Excreta disposal			
Toilet or latrine	85 (57.1%)	136 (59.9%)	
Open field	64 (42.9%)	91 (40.1%)	0.581
Birth order			
1st–3rd	58 (38.9%)	78 (34.4%)	
≥ 4th	91 (61.1%)	149 (65.6%)	0.368
Maternal allergic diseases			
No	60 (40.3%)	162 (71.4%)	
Yes	89 (59.7%)	65 (28.6%)	< 0.001
Maternal smoking			
No	118 (79.2%)	193 (85.0%)	
Yes	31 (20.8%)	34 (15.0%)	0.144
Skin prick reaction			
Any allergen (dust mite or cockroach)			
Negative (< 3 mm)	120 (80.5%)	198 (87.2%)	
Positive (≥ 3 mm)	29 (19.5%)	29 (12.8%)	0.079
Dust mite	21 (14.1%)	18 (7.9%)	0.055
Cockroach	10 (6.7%)	15 (6.6%)	0.969
Grass mix	1 (0.7%)	3 (1.3%)	0.548
Cat	2 (1.3%)	1 (0.4%)	0.336
Dog	1 (0.7%)	0 (0.0%)	0.216
Fungi	0 (0.0%)	0 (0.0%)	
Alternaria	0 (0.0%)	1 (0.4%)	0.417
Allergen specific IgE (asIgE)			
Any asIgE (dust mite or cockroach)			
Negative (< 0.70 kU/L)	100 (67.1%)	171 (75.3%)	
Positive (≥ 0.70 kU/L)	49 (32.9%)	56 (24.7%)	0.082
Any asIgE levels (dust mite or cockroach)			
< 0.70 kU/L	100 (67.1%)	171 (76.2%)	
0.70–3.49 kU/L	25 (16.8%)	42 (18.5%)	
≥ 3.50 kU/L	24 (16.1%)	14 (6.2%)	0.007
IgE to dust mite (≥ 0.70 kU/L)	34 (22.8%)	38 (16.7%)	0.143
IgE to cockroach (≥ 0.70 kU/L)	26 (17.5%)	31 (13.7%)	0.316
Anti-*Ascaris* IgE (≥ 0.70 kU/L)	115 (77.2%)	130 (57.3%)	< 0.001
Exercise-Induced Bronchospasm [14]			
No	121 (85.8%)	213 (96.4%)	
Yes	20 (14.2%)	8 (3.6%)	< 0.001
Geohelminth infections [13]			
Any geohelminth	64 (74.4%)	65 (73.0%)	0.835
*Ascaris lumbricoides*	65 (45.8%)	105 (47.5%)	0.746
*Trichuris trichiura*	82 (57.8%)	129 (58.4%)	0.906
Hookworm	9 (6.3%)	20 (9.1%)	0.352
*Strongyloides stercoralis*	0 (0.0%)	1 (0.5%)	0.422

* Comparison of cases and control subjects: X^2^ or Fisher's exact test.

Number of missing values are given in parentheses.

Exercise-Induced Bronchospasm: 10% fall in FEV_1_ after exercise.

### Effect of age on associations between wheeze and atopy

**Figure 1:**
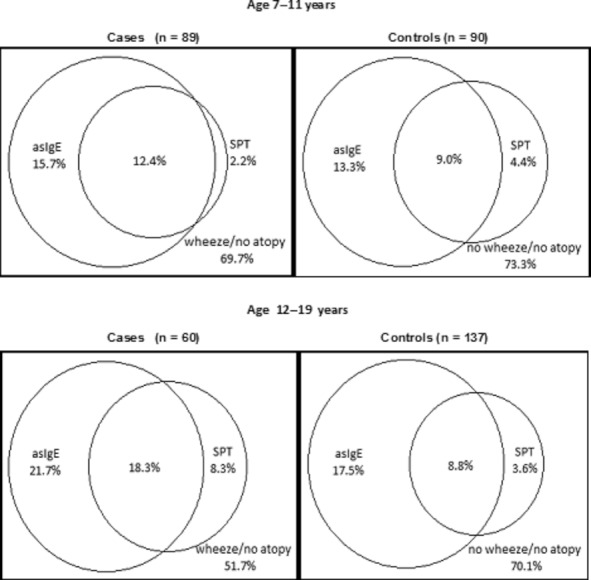
Proportional Venn Diagrams for the overlap between allergen specific IgE (asIgE ≥ 0.70 kU/L) and allergen skin prick test (SPT) responses in cases and controls by age grouping (*N* = 376). Within each diagram, the circles show the proportions of cases or controls with asIgE or SPT responses or where the circles overlap, the proportions with both atopic markers. Areas within the diagram but outside the circles represent the proportions with no positive atopic marker (no atopy) for either cases (wheeze) or controls (no wheeze).

The proportional Venn diagram (Figure 1) showed that a substantial and similar proportion of children aged 7–11 years with (cases) and without (controls) recent wheeze (69.7% vs. 73.3%, respectively) had no evidence of atopy (as measured using either SPT or asIgE to *D. pteronyssinus* and *P. americana*). Among adolescents aged 12–19 years, 51.7% of cases and 70.1% of controls had no evidence of atopy. The second most frequent category for both cases and controls was asIgE (in the absence of SPT), and this proportion increased with age in cases (i.e. 7–11 years, 15.7% vs. 12–19 years, 21.7%) and controls (7–11 years 13.3% vs. 12–19 years, 17.5%). There was a greater overlap of the two markers of atopy among cases than controls in both age groups. Relatively low concordances were observed between the two markers of atopy in cases and controls (*κ* = 0.423 and 0.363 respectively). Among all children, only 44.9% (22/49) of cases with asIgE had a positive SPT, while 75.9% (22/29) of cases with positive SPT had asIgE. The respective proportions among controls were 35.7% (20/56) and 69.0% (20/29) respectively. The associations between markers of atopy and recent wheeze by age grouping are shown in Table 2. The prevalence of SPT was associated with an increased risk of wheeze in the older but not in the younger age group (adj. OR 2.99, 95% CI 1.31–6.80, *P* = 0.009 vs. adj. OR 1.20, CI 0.48–2.99, *P* = 0.697). In the case of specific allergens, SPT to *D. pteronyssinus* was significantly associated with wheeze in the older age group (adj. OR 2.76, 95% CI 1.07–7.13, *P* = 0.036), but there was no association between SPT to *P. americana* and wheeze in either age group. An association between wheeze and asIgE was observed in the older age group only among children with high levels of asIgE (≥3.5 kU/L) (adj. OR 3.90, 95% CI 1.39–10.96, *P* = 0.010). For specific allergens, IgE to *D. pteronyssinus* and *P. americana* was not significantly associated with wheeze in either age group. There was no evidence for a statistically significant interaction between age and atopy on the risk of wheeze (*P*-values for interaction for SPT and asIgE, 0.368 and 0.234 respectively). The presence of specific IgE to *A. lumbricoides* was significantly associated with wheeze in both age groups (7–11 years, adj. OR 2.06, 95% CI 1.05–4.03, *P* = 0.035 vs. 12–19 years, adj. OR 3.00, 95% CI 1.37–6.56, *P* = 0.006). There was no difference in the prevalence of *A.lumbricoides* or other geohelminths between cases and controls in either age group (data not shown).

**Table 2:** Associations of recent wheeze (cases) with allergen specific IgE (asIgE) and skin prick test reactivity (SPT) to house dust mite and cockroach and anti-*Ascaris* IgE

	7–11 years (*n* = 179)			12–19 years (*n* = 197)			Total (*N* = 376)			
				
Variables	*N*	Wheeze *n*(%)	OR (95% CI)*	*N*	Wheeze *n*(%)	OR (95% CI) *	*N*	Wheeze *n*(%)	OR (95% CI) *	PAF (%)
Any asIgE levels (kU/L)										
< 0.70	134	64 (48.1)	1.0	137	36 (26.3)	1.0	273	100 (36.6)	1.0	
0.70–3.49	26	12 (46.2)	0.75 (0.30–1.86)	41	13 (31.7)	1.22 (0.55–2.72)	65	25 (38.5)	1.00 (0.54–1.81)	0.80
			*P* = 0.534			*P* = 0.620			0.979	
≥ 3.50	19	13 (68.4)	1.92 (0.64–5.75)	19	11 (57.9)	3.80 (1.35–10.76)	38	24 (63.2)	2.84 (1.34–6.06)	10.44
			*P* = 0.242			*P* = 0.011			0.007	
Any asIgE (≥ 0.70 kU/L)										
Negative	134	64 (47.8)	1.0	137	36 (26.3)	1.0	271	100 (36.9)	1.0	
Positive	45	25 (55.6)	1.10 (0.53–2.29)	60	24 (40.0)	1.79 (0.91–3.52)	105	49 (46.7)	1.47 (0.89–2.41)	10.52
			*P* = 0.805			*P* = 0.089			*P* = 0.131	
asIgE to HDM (≥ 0.70 kU/L)										
Negative	152	73 (48.0)	1.0	152	42 (27.6)	1.0	304	115 (37.8)	1.0	
Positive	27	16 (59.3)	1.38 (0.56–3.39)	45	18 (40.0)	1.77 (0.86–3.68)	72	34 (47.2)	1.64 (0.93–2.89)	8.91
			*P* = 0.488			*P*=0.123			*P* = 0.087	
asIgE to cockroach (≥ 0.70 kU/L)										
Negative	151	74 (49.0)	1.0	168	49 (29.2)	1.0	319	123 (38.6)	1.0	
Positive	28	15 (53.6)	0.97 (0.41–2.31)	29	11 (37.9)	1.46 (0.61–3.47)	17	26 (45.6)	1.20 (0.65–2.23)	2.91
			*P* = 0.949			*P* = 0.396			*P* = 0.558	
Anti-*Ascaris* IgE (≥ 0.70 kU/L)										
Negative	67	23 (34.3)	1.0	64	11 (17.2)	1.0	131	34 (26.0)	1.0	
Positive	112	66 (58.9)	2.06 (1.05–4.03)	133	49 (36.8)	3.00 (1.37–6.56)	245	115 (46.9)	2.47 (1.50–4.07)	45.93
			*P* = 0.035			*P* = 0.006			*P* < 0.001	
Any SPT (≥ 3 mm)										
Negative	154	76 (49.4)	1.0	164	44 (26.8)	1.0	318	120 (37.7)	1.0	
Positive	25	13 (52.0)	1.20 (0.48–2.99)	33	16 (48.5)	2.99 (1.31–6.80)	58	29 (50.0)	2.05 (1.11–3.78)	9.97
			*P* = 0.697			*P* = 0.009			*P* = 0.022	
SPT to HDM (≥ 3 mm)										
Negative	162	79 (48.8)	1.0	175	49 (28.0)	1.0	337	128 (38.0)	1.0	
Positive	17	10 (58.8)	1.40 (0.47–4.21)	22	11 (50.0)	2.76 (1.07–7.13)	39	21 (53.9)	2.15 (1.04–4.42)	7.54
			*P* = 0.545			*P* = 0.036			*P* = 0.038	
SPT to cockroach (≥ 3 mm)										
Negative	169	85 (50.3)	1.0	182	54 (29.7)	1.0	351	139 (39.6)	1.0	
Positive	10	4 (40.0)	0.80 (0.19–3.33)	15	6 (40.0)	1.91 (0.61–6.03)	25	10 (40.0)	1.37 (0.56–3.34)	1.81
			*P* = 0.763			*P* = 0.269			*P* = 0.494	

* OR estimated by random effects logistic regression model and adjusted by sex, maternal allergic diseases, maternal smoking.

† OR estimated by random effects logistic regression model and adjusted by age, sex, maternal allergic diseases, maternal smoking.

Any SPT/Any asIgE – SPT or specific IgE for either mite or cockroach.

### Effects of geohelminth infections on associations between atopy and wheeze

Geohelminth infections or exposures may act as effect modifiers of the associations between markers of atopy and wheeze. The presence of active geohelminth infections appeared to reduce the size of the ORs for the associations between SPT and wheeze (Table 3; No geohelminths [negative], adj. OR 3.52, 95% CI 1.05–11.79, *P* = 0.041 vs. any geohelminth [positive] adj. OR 1.59, 95% 0.72–3.54, *P* = 0.253), although there was not statistical evidence for interaction (*P* = 0.366). There was some limited evidence also that children with active geohelminth infections had a weaker association between asIgE and wheeze (Table 3). In the case of anti-*Ascaris* IgE, the association between SPT and wheeze did not appear to be affected (Table 3), but there was some evidence that children without anti-*Ascaris* IgE had a stronger association between asIgE and wheeze compared with those with anti- *Ascaris* IgE. Inclusion of anti-*Ascaris* IgE, but not active geohelminth infections, as a variable in the model for the association between asIgE and wheeze (data no shown), resulted in a decrease in OR (without anti-*Ascaris* IgE, adj. OR 1.47, 95% IC 0.89–2.41, *P* = 0.131 vs. with anti-*Ascaris* IgE, adj. OR 1.18, 95% CI 0.70–1.98, *P* = 0.530) suggesting that part of the association may be explained by anti-*Ascaris* IgE.

**Table 3:** Associations of wheeze with allergen specific IgE (asIgE) and skin prick test reactivity (SPT) to house dust mite and cockroach stratified by presence of the geohelminth infection markers, *Ascaris lumbricoides* and/or *Trichuris trichiura* or anti-*Ascaris* IgE

	*A. lumbricoides* and or *T. trichiura* infection					
	
	Negative (*n*=112)			Positive (*n*=251)		
		
	Wheeze *n*(%)/*N*	OR (95% CI)	*P-*value	Wheeze *n*(%)/*N*	OR (95% CI)	*P*-value
Any asIgE levels (kU/L)						
< 0.70	23 (31.9)/72	1.0		73 (38.6)/189	1.0	
≥ 0.70	19 (47.5)/40	2.23 (0.81–6.16)	0.123	27 (43.6)/62	1.05 (0.56–1.98)	0.870
Any SPT						
Negative	29 (33.3)/87	1.0		85 (38.8)/219	1.0	
Positive	13 (52.0)/25	3.52 (1.05–11.79)	0.041	15 (46.9)/32	1.59 (0.72–3.54)	0.253

	Anti-*Ascaris* IgE (kU/L)					
	
	< 0.70 (*n* = 131)			≥ 0.70 (*n* = 245)		
		
	Wheeze *n*(%)/*N*	OR (95% CI)	*P*-value	Wheeze *n*(%)/*N*	OR (95% CI)	*P*-value
Any asIgE levels (kU/L)						
< 0.70	28 (24.1)/116	1.0		72 (46.5)/155	1.0	
≥ 0.70	6 (40.0)/15	2.88 (0.84-9.88)	0.093	43 (47.8)/90	1.00 (0.56-1.76)	0.993
Any SPT						
Negative	28 (24.6)/114	1.0		92 (45.1)/204	1.0	
Positive	6 (35.3)/17	2.53 (0.77–8.27)	0.125	23 (56.1)/41	1.96 (0.93–4.12)	0.075

ORs estimated by random effects logistic regression and adjusted by age, sex, maternal smoking and maternal allergic diseases.

### Effect of geohelminth infections on associations between asIgE and SPT

Geohelminth infections or IgE sensitization to *A. lumbricoides* could modify the association between asIgE and SPT. A significantly weaker association between asIgE and SPT was observed among children with anti-*Ascaris* IgE compared with those without (adj. OR 8.80, 95% CI 2.64–29.28, *P* = 0.001 vs. adj. OR 44.19, 95% CI 3.40–373.80, *P* = 0.005; test for interaction, *P* = 0.016), but the presence of active geohelminth infections did not markedly alter this association (Table 4). The association between asIgE and SPT was altered little by controlling for the effects of anti-*Ascaris* IgE (without anti-*Ascaris* IgE, adj. OR 11.03, 95% CI 4.42–27.53, *P* < 0.001 vs. with adj. OR 13.83, 95% CI 4.85–39.42, *P* < 0.001), suggesting that cross-reactivity between asIgE and anti-*Ascaris* IgE is an unlikely explanation for the dissociation between the results of asIgE and SPT.

**Table 4:** Association between allergen specific IgE (asIgE) and skin prick test reactivity (SPT) to house dust mite and cockroach stratified by presence and absence of *A. lumbricoides* and*/*or *T. trichiura* infection or anti-*Ascaris* IgE

	*A. lumbricoides* and or *T. trichiura* infection					
	
	Negative (*n*=112)			Positive (*n*=251)		
		
	SPT Positive *n*(%)/*N*	OR (95% CI)	*P-*value	SPT Positive *n*(%)/*N*	OR (95% CI)	*P*-value
Any asIgE levels (kU/L)						
< 0.70	6 (8.33)/72	1.0		10 (5.3)/189	1.0	
≥ 0.70	15 (47.5)/40	14.04 (3.44–57.29)	<0.001	22 (35.5)/62	9.16 (2.39–35.07)	0.002

	Anti-*Ascaris* IgE (kU/L)					
	
	< 0.70 (*n* = 131)			≥ 0.70 (*n* = 245)		
		
	SPT positive n(%)/N	OR (95% CI)	*P*-value	SPT positive n(%)/N	OR (95% CI)	*P*-value
Any asIgE (kU/L)						
< 0.70	6 (5.2)/116	1.0		10 (6.5)/155	1.0	
≥ 0.70	11 (73.3)/15	44.19 (3.40–373.80)	0.005	31 (34.4)/90	8.80 (2.64–29.28)	0.001

ORs estimated by logistic regression model with survey estimation and adjusted by age, sex and maternal allergic diseases.

### Associations between markers of geohelminth infection and atopy/wheeze

The associations between geohelminth infection markers and study outcomes are shown in Table 5. Children with anti-*Ascaris* IgE had a higher prevalence of recent wheeze (adj. OR 2.24, 95% CI 1.33–3.78, *P* = 0.003) and asIgE (adj. OR 5.34, 95% CI 2.49–11.45, *P* < 0.001). The effect on wheeze was affected little by adjustment for asIgE (inclusion of asIgE in model, adj. OR 2.13, 95% CI 1.24–3.66, *P* = 0.006), suggesting that the effects of anti-*Ascaris* IgE on wheeze cannot be explained by asIgE. Although anti-*Ascaris* IgE was positively associated with EIB, this effect was not significant (adj. OR 5.03, 95% CI 0.22–112.90, *P* = 0.305). The presence of anti-*Ascaris* IgE was strongly associated with atopic wheeze (i.e. defined by recent wheeze with asIgE ≥ 0.70 kU/L) (adj. OR 6.98, 95% CI 2.69–18.08, *P* = 0.001) and also significantly associated with non-atopic wheeze (adj. OR 2.67, 95% CI 1.48–4.81, *P* < 0.001). On the other hand, active infections with any geohelminth or *A. lumbricoides* alone were not associated with wheeze or atopy, but children with *T. trichiura* infections had a significantly reduced prevalence of atopic wheeze (adj. OR 0.47, 95% CI 0.22–0.98, *P* = 0.043), an observation that was reflected in a strong inverse association between *T. trichiura* infection and the prevalence of SPT (adj. OR 0.42, 95% CI 0.17–0.99, *P* = 0.053).

**Table 5:** Associations of markers of geohelminth infection with wheeze, and presence of skin prick test reactivity (SPT) and allergen specific IgE (asIgE) to house dust mite and cockroach

	Wheeze OR (CI 95%)	SPT (≥ 3 mm) OR (CI 95%)	asIgE OR (CI 95%)	Atopic wheeze OR (CI 95%)	Non-atopic wheeze OR (CI 95%)
Anti-*Ascaris* IgE					
< 0.70 kU/L	1.0	1.0	1.0	1.0	1.0
≥ 0.70 kU/L	**2.24 (1.33–3.78)**	1.15 (0.45–2.95)	**5.34 (2.49–11.45)**	**6.98 (2.69–18.08)**	**2.67 (1.48–4.81)**
	***P***=**0.003**	*P* = 0.761	***P*** **< 0.001**	*P* = 0.001	***P*****< 0.001**
*A. lumbricoides* infection					
Negative	1.0	1.0	1.0	1.0	1.0
Positive	0.90 (0.55-1.48)	0.82 (0.41–1.64)	0.86 (0.53–1.41)	0.76 (0.37–1.55)	0.99 (0.57–1.73)
	*P* = 0.680	*P* = 0.570	*P* = 0.549	*P* = 0.447	*P* = 0.976
*T. trchiura* infection					
Negative	1.0	1.0	1.0	1.0	1.0
Positive	0.72 (0.44–1.18)	0.42 (0.17–0.99)	0.78 (0.47–1.28)	**0.47 (0.22–0.98)**	0.82 (0.46–1.47)
	*P* = 0.187	*P* = 0.053	*P* = 0.318	*P* = 0.043	*P* = 0.510
Geohelminth infections					
Negative	1.0	1.0	1.0	1.0	1.0
Positive	0.94 (0.55–1.62)	0.68 (0.32–1.47)	0.74 (0.44–1.23)	0.49 (0.23–1.04)	1.20 (0.63–2.26)
	*P* = 0.828	*P* = 0.327	*P* = 0.238	*P* = 0.064	*P* = 0.581
Geohelminth ova/anti-*Ascaris* IgE					
Ova -/IgE-	1.0	1.0	1.0	1.0	1.0
Ova +/IgE-	0.80 (0.33–1.91)	0.64 (0.10–3.99)	0.30 (0.04–2.31)	–	1.02 (0.40–2.61)
	*P* = 0.614	*P* = 0.628	*P* = 0.245	–	*P* = 0.961
Ova -/ IgE+	**2.18 (1.08–4.42)**	1.01 (0.40–2.54)	**3.54 (1.48–8.45)**	**4.26 (1.35–13.39)**	**2.58 (1.13–5.91)**
	*P* = 0.030	*P* = 0.982	*P* = 0.005	*P* = 0.013	*P* = 0.025
Ova +/ IgE+	**2.03 (1.01–4.05)**	0.94 (0.40–2.19)	**3.36 (1.53–7.38)**	**3.73 (1.20–11.61)**	**2.39 (1.06–5.38)**
	*P* = 0.046	*P* = 0.885	*P* = 0.003	*P* = 0.023	*P* = 0.036

Statistically significant (*P* < 0.05) ORs are shown in bold. All wheeze outcomes refer to wheeze within the previous 12 months (recent wheeze). Geohelminth ova or infections represent presence of ova of *A. lumbricoides* and/or *T. trichiura*. OR for 4-way analysis of Ova/IgE were adjusted for *a priori* confounders only. ORs for two-level geohelminth exposures were adjusted for the following confounders: wheeze – age, sex, maternal allergic disease, maternal smoking, maternal education, family income, birth order, excreta disposal, dog inside house currently and recent anthelminthic treatment; atopic markers – age, sex, maternal education, family income, birth order, excreta disposal and recent anthelminthic treatment. Atopic wheeze was defined as wheeze within the previous 12 months in the presence of ≥ 0.70 kU/L of house dust mite and or cockroach.

To explore the effects of active infections vs. IgE sensitization to *Ascaris* on study outcomes, we stratified the children into four infection groups according to the presence and absence of geohelminth infections and anti-*Ascaris* IgE (Table 5). The four-group stratification showed significant associations between the presence of anti-*Ascaris* IgE and recent wheeze, the presence of asIgE, and atopic and non-atopic wheeze (Table 5) that was independent of the presence of geohelminth ova in stool samples. No significant associations with study outcomes were observed among children without anti-*Ascaris* IgE.

## Discussion

Childhood asthma has emerged as a major public health problem in many Latin American countries 1, although there are marked differences in asthma prevalence and morbidity between and within countries, particularly between urban and rural populations 24, 25. The causes of regional differences in asthma prevalence are likely to reflect distinct patterns of environmental exposures interacting with genetic factors. Atopy is considered an important determinant of the prevalence 14 and severity 26 of childhood asthma in industrialized countries; however, population-based studies of children and adolescents aged 4–16 years with asthma symptoms 18, 27, 28 and bronchial hyperresponsiveness 29 in Latin America have shown only weak associations with atopy. Thus, the factors that modify the association between atopy and asthma and that affect the prevalence of atopy in Latin American populations are of public health relevance.

Over recent years, there has been interest in the role of geohelminth infections in affecting atopy and asthma prevalence although the findings of studies have been inconsistent 19. In the present case-control analysis, we explored the effects of age and geohelminth infections on the association between atopy and recent wheeze in school-age children living in a rural area of Ecuador. Less than a third of children with wheeze had evidence of atopy to the dominant sensitizing allergens in our study population, house dust mite and cockroach, whereas this proportion increased to half among adolescents. Further, the association between atopy and wheeze was stronger in adolescents compared with children. IgE sensitization to *Ascaris* but not active geohelminth infections (measured by the presence of eggs in stool samples) increased the risk of wheeze in children and adolescents independently of asIgE. The fraction of wheeze that was attributable to anti-*Ascaris* IgE in our study population was 45.9%, while the attributable fractions for asIgE and SPT were just 10.5% and 10.0% respectively. These data strongly support an important role for allergic sensitization to *Ascaris* as a risk factor for wheeze.

Asthma during childhood is complex and has different characteristics at different ages, perhaps as a consequence of the influence of environmental exposures, that may vary by age, on underlying genetic susceptibilities 30. Cohort studies have demonstrated that transient wheeze and non-atopic wheeze phenotypes predominate during the first years of life and mid-childhood respectively. Persistent wheeze, more commonly associated with atopy and airways hyperresponsiveness, tends to appear in late childhood and early adolescence and may continue into adulthood 7. In our study population, there was some evidence that the association between wheeze and atopy increased with age. Previous studies in children and adolescents in underprivileged populations in Latin America have shown weak associations between atopy and wheeze/asthma 2, 18, 27, 28, while a study in adults 31 showed a moderate to high association indicating that the age of the study population could be a possible explanation for differences between studies. Genetic studies have addressed the influence of age on the development of atopy and asthma 6, and have identified distinct susceptibility genes that are associated with childhood and adult asthma 32, 33. Adolescence is characterized by rapid hormonal, physical and behavioural changes, all of which may affect the natural course of asthma. Sex hormones can alter β2-adrenergic responsiveness 34 and female hormones have been shown to increase the production of Th2-like cytokines from peripheral blood mononuclear cells 35. Leptin has been proposed as one of the signals controlling sexual maturation 36 and may also be involved in the regulation of respiratory function 37. Exposure to important environmental exposures and the effects of these on asthma phenotypes may vary by age 11.

The low PAF values for wheeze with respect to atopy observed here (10.5% for asIgE and 10.0% for SPT) are consistent with the findings of the ISAAC phase II study that estimated PAFs for SPT of only 11% for both South American study centres 14. A possible explanation for the low association between markers of atopy and wheeze is exposure to factors that reduce atopy such as chronic helminth infections that are potent regulators of immune function 1, 20. There was some evidence of a weaker association between both markers of atopy and wheeze among children with active geohelminth infections compared with those without (SPT, OR 3.52 vs. OR 1.59; asIgE OR 2.23 vs. OR 1.05 respectively). Geohelminth infections with a pulmonary phase of larval migration of which *A. lumbricoides* is the most relevant – the others being hookworm and *S. stercoralis* that were of low prevalence in our population – may, during chronic infections, attenuate the effects of atopy on pulmonary inflammation thus reducing wheeze symptoms. The exclusively enteric parasite, *T. trichiura,* may also have effects on inflammatory responses at distal sites 38, 39.

In this study, anti-*Ascaris* IgE explained almost half our cases of wheeze and this effect was independent of asIgE, while active geohelminth infections did not appear to affect the prevalence of wheeze. The presence of anti-*Ascaris* IgE has been identified as a risk factor for asthma and wheeze 40–42, and bronchial hyper-reactivity 43–45 in previous studies. A possible explanation could be cross-reactivity between allergens of *A. lumbricoides* and invertebrates such as cockroach and house dust mite. Cross-reactivity would be suggested to be present if the effect of anti-*Ascaris* IgE on wheeze disappeared when controlling for asIgE (i.e. the effect of anti-*Ascaris* IgE on wheeze is considered to be mediated by asIgE). This appeared to be the case in a previous study that demonstrated a strong association between asthma and anti-*Ascaris* IgE in urban Costa Rica where the prevalence of geohelminths was low 45. However, in another study conducted in rural Bangladesh 41, anti-*Ascaris* IgE was shown to be a risk factor for wheeze after adjusting for anti-mite IgE in a population with a high prevalence of geohelminths. Similarly in this study, although anti-*Ascaris* IgE was strongly associated with asIgE, controlling for asIgE had no effect on the association between anti-*Ascaris* IgE and wheeze, indicating that the effect of anti-*Ascaris* IgE on wheeze was independent of asIgE.

There are limited data on the association between markers of geohelminth infections and atopic and non-atopic asthma/wheeze. Our data, from an area of high geohelminth prevalence, showed active geohelminth infections were not associated with wheeze, but that anti-*Ascaris* IgE was a significant risk factor for both atopic and non-atopic wheeze. Previous studies conducted in areas of low geohelminth prevalence have shown the presence of anti-*Ascaris* IgE, that may be the only marker indicating the presence of geohelminth infections 45, to be associated with atopic 40, 44, 45 and non-atopic wheeze and asthma 27, 40. Thus, active infections with *A. lumbricoides* (in low prevalence areas) and the presence of anti-*Ascaris* IgE (irrespective of geohelminth prevalence) may be associated with increased inflammation in the lungs causing wheeze/asthma. The effect of *A. lumbricoides* exposures on wheeze symptoms may vary by the prevalence of infection: in communities with a low prevalence of infection, the presence of active infections with this parasite has been associated with more frequent symptoms of wheeze 19, 27, 46, while in areas of high prevalence active infections do not appear to be associated with the prevalence of wheeze 18. Possible explanations for the association between anti-*Ascaris* IgE and wheeze include: (1) Individuals with an atopic predisposition are more likely to respond to *Ascaris* exposures with an IgE response and are also at increased risk of recent wheeze. In this study, we observed a similar association in atopics and non-atopics although the association was stronger in atopics (2). The presence of anti-*Ascaris* IgE may represent stronger protective immunity to *Ascaris* 47. Such protective immune responses could cause pulmonary inflammation and wheeze when targeted against *Ascaris* larvae migrating through the lungs 19. As protective immunity against ascariasis in humans is not sterile, such an effect would explain why the association between anti-*Ascaris* IgE and wheeze was independent of the presence of eggs in stool samples. (3) The presence of anti-*Ascaris* IgE may be a marker for exposures to closely related zoonotic infections such as *A. suum* and *Toxocara canis* with which *A. lumbricoides* shares extensive immunological cross-reactivity 48. Human infections with *Toxocara* and *A. suum*, causing visceral larva migrans, have been associated with asthma-like symptoms 49, 50, 51. Overall, our observations point to the importance of the immunological response to *A. lumbricoides* rather than the presence *per se* of geohelminths, as an important determinant of wheeze in our study population.

There are several limitations to this analysis that should be considered. Firstly, we defined atopy to just two dominant aeroallergens, house dust mite and cockroach. In our study population, house dust mite and cockroach are the most important environmental allergens when atopy is measured either by SPT or asIgE. An analysis of SPT responses to seven relevant aeroallergens in the cross-sectional study of 3960 children in which the present case-control analysis was nested showed that only 3.1% of children with negative SPTs to mite and cockroach had positive SPTs to any of the other allergens tested 18. These results are consistent with those of previous studies from school children in Ecuador 52, 53. With respect to asIgE, measurements were restricted to *D. pteronyssinus* and *P. americana* based on the findings of previous surveys conducted in similar populations in Ecuador 15, 54. A previous analysis of 50 plasma samples from school children living in the study area of specific IgE for *D. pteronyssinus*, *P. americana*, rye grass, cat, and *Aspergillus* showed that none of the children negative for *D. pteronyssinus* and *P. americana* were positive for any of the other allergens (Cooper PJ et al., unpublished data). Similarly, the tropical mite, *Blomia tropicalis*, does not appear to be an important sensitizing mite in our population – a previous survey in the same population showed a very low prevalence of SPT to this mite (1.2%) and an analysis of mites and mite antigens in dust samples showed that > 90% of mites were *D. pteronyssinus* and few dust samples had measurable levels of Blo t 5, the dominant *B. tropicalis* allergen (Cooper PJ et al., unpublished data). We can be confident that we correctly identified most children with atopy. We used a higher cut-off of ≥ 0.70 kU/L for positivity of tests for specific IgE rather than the conventional cut-off of ≥ 0.35 kU/L because of the possibility of false-positive reactions at low titre that may be associated with the extensive immunological cross-reactivity between helminth allergens and aeroallergens 55, 56. Secondly, we used recent wheeze as a proxy for asthma. Wheeze cases and non-wheeze controls were identified from parental questionnaires based on that used in the ISAAC Phase 2 study 22 in which children with wheeze within the previous 12 months were identified and their status was confirmed by repeating the same questions approximately 2 years later. Viral respiratory tract infections (RTIs) are a frequent cause of wheeze and are the major cause of asthma exacerbations in children 57, and viral RTIs are likely to be a significant cause of wheeze in our study population. In this study, cases represented persistent wheeze (at least one episode of wheeze in the previous year on two occasions), which may be a better proxy for asthma. EIB was used as a marker of the presence of bronchial hyper-reactivity (BHR) at the time of evaluation. Relatively, few wheeze cases had a positive exercise test (14.2%) suggesting either that wheeze in our population is mild and is not associated with significant BHR or that this test may not be a good measure of BHR in the conditions of high ambient humidity 58 present throughout the year in the study area. Thirdly, given the sample size, we had limited power for some of the comparisons done, and cannot exclude the possibility of type 2 statistical errors. However, we were able to detect significant effects for the primary study exposures (age and geohelminth exposures) and thus had sufficient power for these associations. Lack of power may be a particular issue in this study where tests for interaction were done – only one of the interactions tested was statistically significant. Fourthly, we did not measure acetaminophen use in this study, an exposure that has been suggested recently to have a role in the development of childhood asthma 59, and cannot exclude residual confounding by acetaminophen use. Limited access to health care and a lack of community pharmacies in the study area may minimize such bias. Finally, the case-control design of the study limits our ability to infer causality or the direction of effects between presumed exposures and outcomes.

In conclusion, our data from a case-control study of wheezing illness in a rural area of tropical Ecuador has provided evidence that most wheeze was not attributable to atopy to house dust mite and cockroach, although the association between atopy and wheeze did appear to increase with age from childhood to adolescence. We identified the presence of anti-*Ascaris* IgE as an important risk factor for wheeze and this association appeared to be independent of asIgE, and was observed for both atopic and non-atopic wheeze. Active geohelminth infections were not associated with wheeze although there was some evidence that active infections reduced the association between atopy and wheeze. Future studies will be necessary to identify the mechanisms by which anti-*Ascaris* IgE may increase the prevalence of wheeze and could provide novel targets for interventions either for the primary prevention or treatment of asthma in childhood.
